# Skeletal Muscle Pump Drives Control of Cardiovascular and Postural Systems

**DOI:** 10.1038/srep45301

**Published:** 2017-03-27

**Authors:** Ajay K. Verma, Amanmeet Garg, Da Xu, Michelle Bruner, Reza Fazel-Rezai, Andrew P. Blaber, Kouhyar Tavakolian

**Affiliations:** 1 Department of Electrical Engineering, University of North Dakota, Grand Forks-58202, USA; 2 Department of Engineering Science, Simon Fraser University, Burnaby-V5A 1S6, Canada; 3 Department of Biomedical Physiology and Kinesiology, Simon Fraser University, Burnaby-V5A 1S6, Canada

## Abstract

The causal interaction between cardio-postural-musculoskeletal systems is critical in maintaining postural stability under orthostatic challenge. The absence or reduction of such interactions could lead to fainting and falls often experienced by elderly individuals. The causal relationship between systolic blood pressure (SBP), calf electromyography (EMG), and resultant center of pressure (COPr) can quantify the behavior of cardio-postural control loop. Convergent cross mapping (CCM) is a non-linear approach to establish causality, thus, expected to decipher nonlinear causal cardio-postural-musculoskeletal interactions. Data were acquired simultaneously from young participants (25 ± 2 years, n = 18) during a 10-minute sit-to-stand test. In the young population, skeletal muscle pump was found to drive blood pressure control (EMG → SBP) as well as control the postural sway (EMG → COPr) through the significantly higher causal drive in the direction towards SBP and COPr. Furthermore, the effect of aging on muscle pump activation associated with blood pressure regulation was explored. Simultaneous EMG and SBP were acquired from elderly group (69 ± 4 years, n = 14). A significant (p = 0.002) decline in EMG → SBP causality was observed in the elderly group, compared to the young group. The results highlight the potential of causality to detect alteration in blood pressure regulation with age, thus, a potential clinical utility towards detection of fall proneness.

Stable upright posture is achieved through numerous complex physiological interactions^1^. Such interactions, through intersystem information exchange, assure a stable upright posture under orthostatic stress. Standing causes redistribution of blood (blood pooling) in the compliant distensible veins of the lower body (mostly in pelvic and leg) regions as a consequence of gravity. The consequent reduction in central venous pressure leads to decline in venous return and stroke volume. The baroreflex mediated vagal withdrawal and activation of the sympathetic nerve, leading to increased heart rate and systemic vascular resistance^2^ is a typical compensatory reflex mechanism to a reduction in stroke volume. The baroreflex mechanism helps restore cardiac output and blood pressure, however, this blood pressure regulation mechanism lasts only for a few beats. With drain out of the pulmonary reservoir, the baroreflex mechanism has shown limited effect towards blood pressure regulation under orthostatic challenge, owing to a small increase in venous return^3^. During upright posture, skeletal muscles help maintain venous return and consequently cardiac output by compressing underlying veins in order to increase blood flow back to the heart (skeletal muscle pump). Thus, the role of lower leg muscles (calf skeletal muscles) in regulating blood pressure homeostasis while attaining upright posture is indispensable^4^. The absence of such pumping while assuming upright posture could lead to a substantial drop in blood pressure (orthostatic hypotension). Orthostatic hypotension is quite prevalent among elderly, patients with neurodegenerative diseases, and in astronauts, therefore, making it one of the major causes of fall in such population groups^5^,^6^,^7^,^8^,^9^,^10^,^11^,^12^.

Postural stability is dependent on input from visual, cognitive, somatosensory, and vestibular systems^11^,^13^,^14^. In the absence of such input, an alteration in postural stability along with blood pressure and muscle activity is observed^15^. This is indicative of inter-relationship between cardio-postural-musculoskeletal systems^16^. This inter-relationship has been recently validated during quiet standing by exhibiting the presence of significant coherence (linear coupling) between variables belonging to respective systems^15^,^16^,^17^. However, these studies did not investigate cause and effect relationship between the variables pertaining to such systems. While the mechanical effects of skeletal muscle pump on blood pressure were well studied^18^,^19^,^20^, quantified knowledge of causal drive from skeletal muscles towards blood pressure, and the causal relationship in the reverse direction, which is hypothesized as the muscle pump activation mediated by baroreflex in response to blood pressure alteration, is unknown.

Causality describes the directional relationship between cause and its effect. The human body as a highly integrated group of dynamic and adaptive systems often shows cause and effect relationships, such relationship has been studied in neural^21^,^22^,^23^, cardiovascular^24^,^25^, cardio-respiratory^26^,^27^, and cardio-neural systems^28^,^29^. The established knowledge of causality between dynamical systems could be exploited for physiological system performance monitoring, as; a significant deviation in the behavior of causally linked systems from the established norm could be symptomatic of system impairment. To this end, the commonly applied Granger causality method measures the ability of one signal to predict the future of other to establish causal behavior. However, assumptions of linear statistical inference, stationary signal behavior, and determination of appropriate model order limit the application of Granger causality methods to linear and stationary systems^21^,^22^. Transfer entropy, a nonlinear model free methodology is often considered for addressing limitations of Granger causality^30^,^31^,^32^. Nevertheless, the assumption of stationary signal behavior and the requirement to estimate probability density function of signals under consideration limit its application^33^. Physiological signals are inherently nonlinear in nature, thus, a nonlinear approach would be necessary to obtain accurate inference with respect to the dynamics of the complex causal interplay between physiological systems.

Convergent cross mapping (CCM), a nonlinear approach for estimating causality between two time series is based on the state space reconstruction of a time series called ‘shadow-manifold’^34^. Causality is estimated by quantifying the correspondence between two manifolds. State space reconstruction of variables is dependent on the selection of time delay (τ) and embedding dimension of reconstruction (E)^35^,^36^. Under optimal choice of two parameters, CCM is expected to uncover accurate underlying nonlinear directional physiological interaction. Moreover, in contrast to Granger causality, the CCM method is capable of inferring causality in systems with weak to moderate coupling, while the performance of Granger causality is contingent on data separability^35^,^37^. Causality analysis using Granger-based approaches is often subject to statistical hypothesis testing, whereby, causality inference is based on acceptance/rejection of the null hypothesis while the physiological systems are interacting continuously with variable intensity.

On the other hand, CCM infers causality in terms of strength of coupling, thus, revealing vital information regarding the degree to which the interacting variables are coupled. The quantified knowledge of the strength of directional interaction can have a clinical relevance; as aging or pathology may cause an alteration in the strength of such interaction. The strength of the CCM method has been demonstrated in physiological applications to understand blood pressure and cerebral blood flow velocity interaction^38^ and in the interaction between heart rate variability and the electroencephalographic signals^39^. Additionally, the performance of CCM method has been shown to be superior to the Granger causality with signals of nonlinear nature^39^. With evidence of success in the literature, CCM is expected to accurately unearth dynamics of underlying physiological interactions between cardio-postural-musculoskeletal systems (cardio-postural control loop), a pivotal intersystem interaction required for maintaining stable upright posture.

To the best of our knowledge, our preliminary study is the only effort so far in the direction of establishing causality in the cardio-postural control loop^40^,^41^,^42^,^43^. The results obtained from small sample size (n = 5 or 7) hinted towards the existence of bidirectional (feedback) causality between such systems. In current work, we study the CCM method for its suitability to investigate causal relationship in the cardio-postural control loop. Additionally, we thoroughly study the free parameters of choice, i.e., time delay and embedding dimension of reconstruction to ascertain the optimal combination to establish the cardio-postural-musculoskeletal interactions. Further, we study a larger sample size (n = 18) of young and healthy participants to generalize the existence of causal interaction in cardio-postural-musculoskeletal systems via a controlled sit-to-stand experimental protocol, which has been used in the literature to induce orthostatic challenge^15^,^44^. Lastly, we explored the sensitivity of the CCM method towards discriminating the behavior of skeletal muscle pump mediated blood pressure regulation (EMG → SBP) and baroreflex mediated skeletal muscle activation (SBP → EMG) due to aging. In this research, we hypothesize to observe strong muscle pump-blood pressure (EMG ↔ SBP), postural sway-muscle pump (COPr ↔ EMG), and postural sway-blood pressure (COPr ↔ SBP) causal interactions to regulate blood pressure in response to the orthostatic challenge. The blood pressure regulation through heart rate was also assessed by calculating arterial baroreflex sensitivity (BRS) of the study participants of two groups to understand alteration in the BRS with respect to causality due to aging.

## Methods

### Data Collection

Data was acquired from 18 healthy young (8 females; age: 25 ± 2 years; height: 174 ± 8 cm; weight: 68 ± 11 kg) and 14 healthy elderly (8 females; age: 69 ± 4 years; height: 165 ± 13 cm; weight: 66 ± 17 Kg) participants during a 10-minute sit-to-stand test. Participants under medication that could alter cardiovascular and postural stability along with those with any history of cardiovascular, respiratory, neurological, and major musculoskeletal injuries or hormone imbalance were screened out from the experimentation. The sit-to-stand test required participants to be seated for 5 minutes, after this, participants were passively assisted to standing phase to maintain a quiet stance for additional 5 minutes. During a 10-minute period electrocardiogram (ECG), blood pressure, calf EMG and COP signals were simultaneously acquired at a sampling rate of 1000 Hz using National Instruments (National Instruments Inc, TX, USA) data acquisition system. For the elderly group, COP signal was not a part of the ethics, hence, it was not acquired.

ECG was acquired in a standard lead II electrode configuration using LifePak 8 (Medtronic Inc, MN, USA). Continuous blood pressure was recorded non-invasively using finger photoplethysmograph cuff from Finometer model 1 (FMS, Amsterdam, The Netherlands). Medio-lateral (COPx) and anterior-posterior (COPy) center of pressure signals were derived from force and moment data obtained using force platform (Accusway Plus, AMTI, USA). Calf EMG was acquired from four different leg muscles namely tibialis anterior, lateral gastrocnemius, medial gastrocnemius, and medial soleus using the 8 channel EMG acquisition system Bangoli-8 (Delsys Inc, MA, USA) in the form of transdermal differential recordings.

The study was approved to be of minimal risk by the research ethics board of Simon Fraser University (SFU). Data acquisition protocols were performed according to relevant guidelines and regulations set by research ethics board of SFU. Participants had the option to terminate experiment any time. Written informed consent was obtained from each participant before experimentation. All participants were required to abstain from alcohol, exercise, and ingesting caffeine 24 hours before the experiment. The data acquisition was performed at the Aerospace Physiology Laboratory, Department of Biomedical Physiology and Kinesiology, SFU.

### Convergent Cross Mapping

Using the CCM method, for Y having a causal influence on X (Y → X), causality is inferred by quantifying the degree to which historical records of X can be used to accurately estimate the states of Y. In order to do so, first, the shadow (reconstructed) manifold of Y(M_Y_) and X(M_X_) are constructed using lagged coordinates of variables Y and X, respectively^34^,^36^. The lagged coordinates of Y, is formed as: 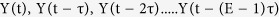; where E and τ are embedding dimension and time lag used for constructing shadow manifold, respectively, the reconstructed Y range from t = 1 + (E−1)τ to t = L, where L is data length. Similarly, the lagged coordinates of X are formed. Next, a minimum of E + 1 nearest neighbors found on M_X_ are used to find the neighbors on M_Y_ to estimate Y. More detailed algorithmic explanation of the steps to perform a CCM causality is explained in the supplementary material of Sugihara *et al*.’s work^34^. The estimated Y using M_X_ is denoted as 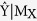. Once estimates of Y are determined, the strength of causality flowing from Y to X is quantified by calculating the Pearson correlation coefficient (ρ) between the original (reconstructed Y) and estimated Y. Mathematically, 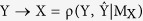.

Using CCM method unidirectional and bidirectional causality can be detected. In the case of a unidirectional causality (X → Y), the driver X can be estimated using a historical record from M_Y_. However, Y cannot be estimated using the historical record from M_X_. The strength of causality varies between 0 and 1, where 0 represents the absence of causality and 1 represents maximum causality. Therefore, the ideal case of unidirectional causality (X → Y) could be represented as; 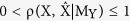 and 
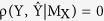
. In the case of bidirectional causality (X → Y and Y → X) the historical record from M_Y_ can be used to estimate X and historical record from M_X_ can be used to estimate Y. In this case, both the correlation of X and estimated X as well as the correlation of Y and estimated Y will vary between 0 and 1. Mathematically, 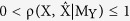 and 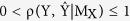. However, the correlation between the variable and its estimate having a stronger effect on the other variable will converge to a higher correlation coefficient value. For example, if X has a stronger effect on Y compared to vice-versa then X → Y will converge to a higher value than Y → X i.e. 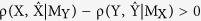. The accuracy of the causality improves with increasing data length marked by increase in correlation between the original and estimated variables (convergence)^34^, also, the notion by which CCM infers causality is contrary to the one proposed by Granger hence it’s termed as cross mapping, as in CCM, response is used to estimate driver^45^.

### Data Processing

All acquired signals were low-pass filtered at a cutoff frequency of 20 Hz. Only standing data was used for studying causality between variables in cardio-postural-musculoskeletal systems. The first minute of data was discarded to eliminate possible motion artifact incorporated in the signals during the sit-to-stand transition. The QRS complex was detected from ECG using the Pan-Tompkins algorithm^46^, R-R time series was obtained as the time difference between two adjacent QRS complex. Beat-by-beat SBP was obtained from continuous finger blood pressure signal by detecting its peak within each R-R interval. SBP time series was then interpolated to 1000 Hz using spline interpolation to create an evenly sampled signal. To represent overall muscle activity in the form of aggregate EMG, the rectified EMG acquired from four different muscles of both legs were added. COPr signal was obtained from medio-lateral (COPx) and anterior-posterior (COPy) center of pressure as a resultant sum of the two vectors^15^. Data were then resampled to 10 Hz before further processing. The resampling of data to a lower sampling rate was based on a previous study where cardio-postural control loop interaction was shown to exist in the lower frequency range (<1 Hz)^15^.

The resampled signals (2 at a time) were input to the CCM algorithm. The correlation coefficient value (magnitude) at which each causal event converged were taken into account as a marker degree of causal information flowing from one variable to another and vice-versa. The statistical significance of correlation was set at α = 0.05. For each analysis, depending upon convergence of causality of the signal pair under analysis, three scenarios were possible: (1) unidirectional (X → Y or Y → X), (2) bidirectional (X → Y and Y → X i.e. X ↔ Y), and (3) no causality. The data analyses were performed in MATLAB (Mathworks Inc, MA, USA).

The Arterial baroreflex sensitivity (BRS), a marker of autonomic blood pressure regulation, was calculated using the sequence method^47^. The BRS computation required R-R intervals (ms), SBP (mmHg) and a threshold as input parameters. A sequence of three or more beats for which R-R and SBP increased or decreased in the same direction with an absolute change in SBP greater than the threshold (1 mmHg) was taken into account^47^.

### Statistical Analysis

The normality of the distribution was checked using Shapiro-Wilk test of normality (IBM SPSS Statistics 23, IBM, USA). Statistical test for significance was conducted using a 2-tailed student t-test for normally distributed data otherwise, Wilcoxon rank sum test was performed, both in MATLAB (Mathworks Inc., MA, USA). A significance level of 1%, 5%, or 10% was considered and it will be mentioned appropriately throughout the text. All results are presented as mean ± standard deviation (SD) unless mentioned otherwise.

## Results

The performance of CCM in effectively revealing causality between two time series data is dependent on the optimal choice of E and τ. The optimal value of E was determined to be 4 using false nearest neighbor (FNN) algorithm^48^. The resampled EMG, COPr, and SBP signals were input to the FNN algorithm. Based on the minimization of average percent of false nearest neighbor, the optimal E was chosen. Next, the optimal time delay was chosen empirically by calculating the EMG ↔ SBP, SBP ↔ COPr, and EMG ↔ COPr causality at different time delay for the chosen embedding dimension of reconstruction (i.e., E = 4). The time delay was varied from 2 to 26 samples in a step of 2 samples. The different causal events attained stability around a delay of 20. Finally, τ = 20 and E = 4 was used for CCM causality analysis between cardio-postural-musculoskeletal systems and all CCM results presented in this article were obtained using such parameters unless mentioned otherwise.

**Figure 1:**
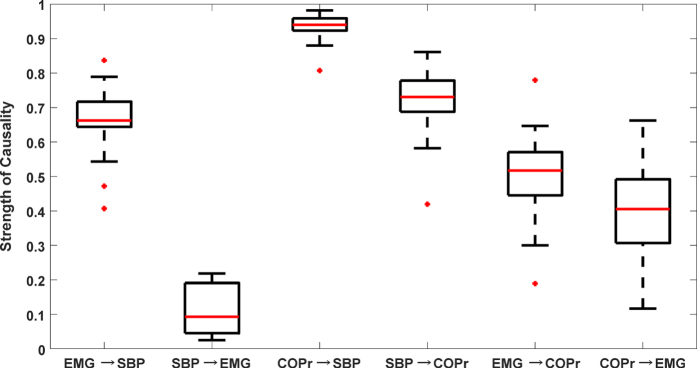
Box plot representations of the strength of causal interaction in the cardio-postural control loop in young participants (n = 18) during a 4 minute of quiet standing. The causality strength of non baroreflex events i.e. EMG → SBP and COPr → SBP were significantly higher (p < 0.001) compared to the strength of baroreflex events i.e. SBP → EMG and SBP → COPr. For EMG ↔ COPr interaction, the causality strength of EMG → COPr was significantly higher (p = 0.06) than COPr → EMG.

As per our hypothesis, the outcome of causality analysis between cardio-postural-musculoskeletal variables resulted in the existence of bidirectional causality. The population-wide behavior of CCM causality between respective signals is summarized in Fig. 1. In the cardio-postural control loop causal interaction, we observed muscle pump mediated blood pressure control (EMG → SBP, 0.66 ± 0.10), baroreflex mediated muscle pump activation (SBP → EMG, 0.11 ± 0.07), muscle pump mediated posture control (EMG → COPr, 0.49 ± 0.15), postural sway mediated muscle pump activation (COPr → EMG, 0.40 ± 0.16), baroreflex mediated posture control (SBP → COPr, 0.71 ± 0.11), and posture mediated blood pressure control (COPr → SBP, 0.93 ± 0.04).

**Figure 2:**
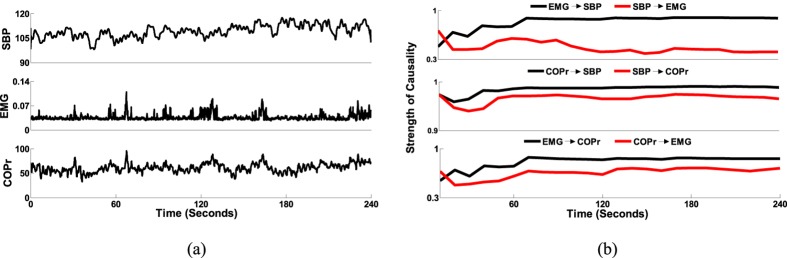
(**a**) An example of simultaneously recorded physiological signals. Systolic blood pressure in mmHg (top), rectified calf electromyography in a.u. (middle), and resultant center of pressure in mm (bottom) during last 4 minutes of quiet standing from one participant (M, age: 28 years, weight: 86 kg, height:177 cm). (**b**) Convergence plot of different causal activities. EMG↔SBP (top), SBP↔COPr (middle) and EMG↔COPr (bottom) computed between the signals in Fig. 2a. The figure indicates EMG → SBP, COPr → SBP, and EMG → COPr to be the dominant direction of causal information flow.

The dominant causal interaction (X → Y or Y → X) was determined by performing a test of significance between forward (X → Y) and reverse (Y → X) causal activities for different interactions (EMG ↔ SBP, EMG ↔ COPr, SBP ↔ COPr). If there existed a significant difference between the two, the difference was considered as an indicator of one system being a dominant driver of the other. To this end, the non-baroreflex events (EMG → SBP and COPr → SBP) were found to be significantly higher (p < 0.001) than the baroreflex events (SBP → EMG and SBP → COPr). The EMG ↔ COPr interaction revealed muscle pump driven (EMG → COPr) posture control to be more dominant (p = 0.06) than the reverse case (COPr → EMG). Figure 2a is an example of simultaneously acquired SBP, EMG, and COPr signals during the last 4-minute period of quiet standing. Figure 2b shows the convergence plot of the CCM causality between respective signals; showcasing dominant EMG → SBP, COPr → SBP, and EMG → COPr activities, discernable by their convergence to a higher correlation coefficient value in comparison to the convergence of causality in the reverse direction i.e. SBP → EMG, SBP → COPr, and COPr → EMG.

**Figure 3:**
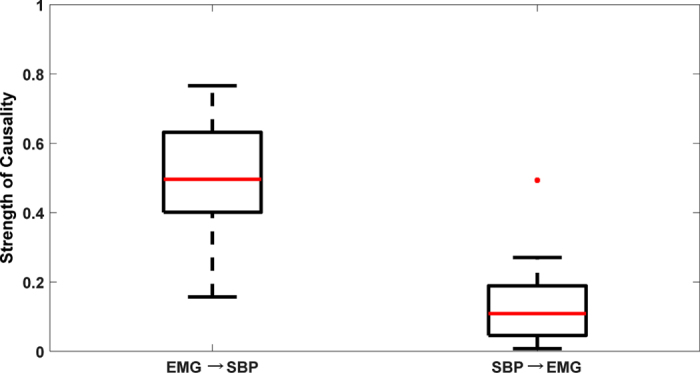
Box plot representation of EMG ↔ SBP causality in the elderly group (n = 14). The non-baroreflex (EMG → SBP) event was found to be significantly higher (p < 0.001) than the baroreflex event (SBP → EMG).

**Figure 4:**
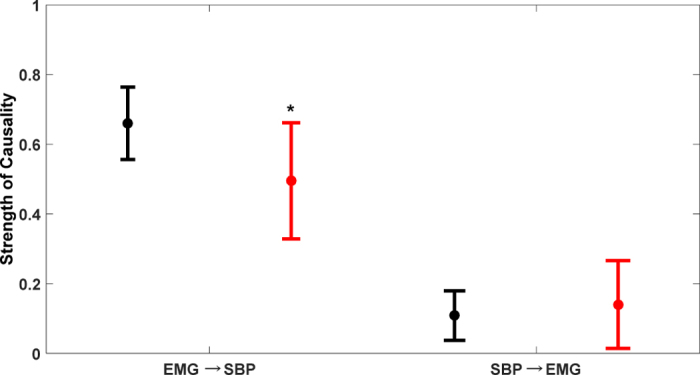
Comparison of EMG ↔ SBP causality (mean ± SD) between two population groups. In elderly group (red) the strength of EMG → SBP causality declined significantly (p = 0.002) while SBP → EMG causality did not change (p = 0.37) compared to young group (black). *Represents significant difference at α = 0.01.

In order to validate the potential of CCM methodology to differentiate causality with age, causality (EMG ↔ SBP) for the elderly group was calculated (Fig. 3) and compared with that of the young group (Fig. 4). In the elderly group, a significant decline in EMG → SBP (0.50 ± 0.17, p = 0.002) activity and no significant change in SBP → EMG (0.14 ± 0.13, p = 0.37) activity were observed compared to the young group (Fig. 4). The arterial baroreflex sensitivity for the two group was significantly different (p < 0.001), with the elderly group (3.63 ± 2.54 ms/mmHg) showing a significant decline in BRS compared to the young group (9.00 ± 3.58 ms/mmHg). One young participant was excluded from the BRS calculation due to an outlier.

## Discussion

Although the role of the interaction between physiological systems is well-known for the maintenance of stable upright posture, the directional information flow between such systems, in order to compensate for external perturbation to achieve postural stability is not generalized. The current work investigated the existence of bidirectional (feedback) causality in cardio-postural-musculoskeletal systems in young, healthy population.

Further differentiation in the strength of muscle pump-baroreflex (EMG ↔ SBP) directional interaction due to aging was explored. The external perturbation to evoke physiological alteration was applied via a controlled sit-to-stand test. The novel insights unearthed by a thoroughly validated causality methodology confirmed previously speculated, yet unclear, directional information flow in the cardio-postural control loop. The advantages of CCM methodology was exploited to quantify the role of the individual physiological link of the cardio-postural control loop (EMG ↔ SBP, SBP ↔ COPr, and EMG ↔ COPr) towards maintaining postural stability. The key finding of the current work was the central role of skeletal muscle pump in facilitating increase in the venous return to the heart and simultaneously controlling the postural sway.

The causal information flow from one physiological system to the other and vice-versa was of variable strength, suggesting one system to be the dominant driver of another system. In general, causal information flow of significantly greater strength (p < 0.001) from skeletal muscle pump to SBP was observed, indicating stronger mechanical skeletal muscle pump effect on blood pressure (EMG → SBP, 0.66 ± 0.10) compared to the baroreflex mediated skeletal muscle activation (SBP → EMG, 0.11 ± 0.07). Similarly, postural sway driven control of SBP (COPr → SBP, 0.93 ± 0.04) was significantly stronger (p < 0.001) than the baroreflex mediated control of postural sway (SBP → COPr, 0.71 ± 0.11) and the muscle pump mediated control of postural sway was stronger (p = 0.06) than the reverse, postural sway mediated skeletal muscle activation.

**Figure 5:**
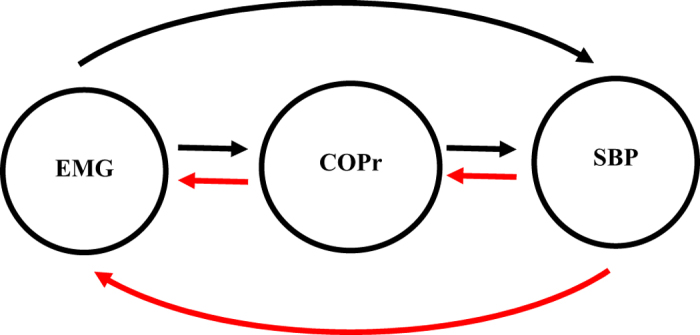
Two primary pathways of causal information flow. Dominant causality (black) from skeletal muscle pump to systolic blood pressure through postural sway and feedback (red) from systolic blood pressure to skeletal muscle pump through postural sway.

This behavior of causality in the cardio-postural control loop highlighted the two-fold role of the skeletal muscle pump; a) driving control of the cardiovascular system by increasing venous return and b) driving control of postural system by controlling postural sway through skeletal muscle activation. There were two primary pathways of causal information flow observed in the cardio-postural control loop, a) stronger non-baroreflex driven control of SBP through postural sway and b) weaker baroreflex mediated activation of skeletal muscles through postural sway. These two pathways manifest the bidirectional transitive behavior (X ↔ Y, Y ↔ Z then X ↔ Z) of the cardio-postural control loop towards the regulation of blood pressure. The two pathways are shown in Fig. 5, the dominant (non-baroreflex mediated) direction of information flow is shown in black while the non-dominant (baroreflex mediated) direction of information flow is shown in red.

To ascertain the potential of CCM causality in differentiating the alterations in the strength of directional information flow between physiological systems due to aging; the established baseline causal behavior of EMG ↔ SBP interaction obtained from the young group was compared with the elderly group. The comparison result highlighted a significant decline from young to elderly group (0.66 ± 0.10 vs 0.50 ± 0.17, p = 0.002) in the strength of skeletal muscle pump driven control of blood pressure (EMG → SBP), while the reverse causality signifying baroreflex mediated skeletal muscle pump activation (SBP → EMG) did not achieve statistical significance (0.11 ± 0.0.07 vs 0.14 ± 0.13, p = 0.37), Fig. 4. This observation accentuated the effect of aging on the skeletal muscles and decline in the ability to facilitate a venous return to the heart while assuming an upright posture, potentially due to loss of muscle mass, therefore, making the elderly population more sensitive to orthostatic hypotension.

Since baroreflex mediated muscle pump activation (SBP → EMG) did not alter significantly with age, it suggests that blood pressure homeostasis is mainly achieved through mechanical muscle pump and baroreflex mediated control has a limited role in driving skeletal muscle activation, this could be further emphasized, by an observation of significant decline (p < 0.001) in the arterial baroreflex sensitivity in the elderly (3.63 ± 2.54 ms/mmHg) compared to young (9.00 ± 3.58 ms/mmHg) group. The findings of this comparison open further avenues regarding the importance of muscle pump in blood pressure regulation. Clinically, this finding can be exploited towards early diagnosis of syncope prone individuals to limit falling incidents. Additionally, it is known that pathologies such as stroke, concussion, and Parkinson’s disease impair skeletal muscle functionality as well as alter postural stability^49^,^50^,^51^, therefore, causality can be advantageous for monitoring effectiveness of treatment towards improving muscle pump functionality as well as monitoring cardio-postural control loop behavior following a pathological event during a rehabilitation.

## Conclusions

The current study has following conclusions. First, an establishment of directional information flow in the cardio-postural control loop, based on data from the young and healthy population. The establishment of causality was achieved by quantifying causality between cardiovascular-postural-musculoskeletal systems by a nonlinear CCM methodology. Second, the non-baroreflex events (EMG → SBP and COPr → SBP) of the cardio-postural control loop have a significantly higher causal drive towards SBP and COPr in comparison to the baroreflex events (SBP → EMG and SBP → COPr) (Fig. 1). We did not observe an alteration in this behavior due to aging (Fig. 3). Third, the study highlighted, the imperative role of skeletal muscles in the regulation of blood pressure and simultaneously driving control of the postural sway, through a significantly higher drive in the direction of blood pressure and postural sway (EMG → SBP and EMG → COPr), importantly, the study accentuates decline in the strength of muscle pump mediated blood pressure control (EMG → SBP) with age. Lastly, the CCM methodology showed the potential of differentiating causality due to aging and thus, can have a clinical applicability in monitoring muscle pump functionality.

### Limitations and Future Work

The limitation of the current study was the unavailability of the center of pressure data (COPr) in the elderly group, as a result, the effect of aging on cardio-postural control loop was not generalized, however, this shall be explored in the future work. Additionally, both population group studied in this research were healthy, therefore, the alteration in causality observed was solely due to aging. The role of skeletal muscles towards blood pressure regulation and the behavior of baroreflex mediated skeletal muscle activation under pathological conditions is still an open field. Future comparison of the cardio-postural control loop generalized in the current research with cardio-postural control loop established under more challenging physiological condition (neural mediated syncope, exercise, and tilt test) and post pathological events (concussion, stroke, and Parkinson’s disease) will further our understanding regarding the behavior of complex directional interaction between physiological systems forming the cardio-postural control loop. Such findings will be a step forward towards clinical applicability of causality established in physiological systems, towards diagnosis and prevention of fall proneness, as well as monitoring the rehabilitation efficacy post pathology.

## Additional Information

**How to cite this article:** Verma, A. K. *et al*. Skeletal Muscle Pump Drives Control of Cardiovascular and Postural Systems. *Sci. Rep.* **7**, 45301; doi: 10.1038/srep45301 (2017).

**Publisher's note:** Springer Nature remains neutral with regard to jurisdictional claims in published maps and institutional affiliations.
